# Follow-Up of Blood Pressure, Arterial Stiffness, and GFR in Pediatric Kidney Transplant Recipients

**DOI:** 10.3389/fmed.2021.800580

**Published:** 2021-12-16

**Authors:** Anna Végh, Adrienn Bárczi, Orsolya Cseprekál, Éva Kis, Kata Kelen, Szilárd Török, Attila J. Szabó, György S. Reusz

**Affiliations:** ^1^First Department of Pediatrics Semmelweis University, Budapest, Hungary; ^2^Medical Imaging Centre Semmelweis University, Budapest, Hungary; ^3^Department of Transplantation and Surgery Semmelweis University, Budapest, Hungary; ^4^Gottsegen National Cardiovascular Center, Budapest, Hungary

**Keywords:** hypertension, arterial stiffness, ABPM, PWV, transplantation

## Abstract

Pediatric renal transplant recipients (RTx) were studied for longitudinal changes in blood pressure (BP), arterial stiffness by pulse wave velocity (PWV), and graft function.

**Patients and Methods:** 52 RTx patients (22 males) were included; office BP (OBP) and 24 h BP monitoring (ABPM) as well as PWV were assessed together with glycemic and lipid parameters and glomerular filtration rate (GFR) at 2.4[1.0–4.7] (T_1_) and 9.3[6.3–11.8] years (T_2_) after transplantation (median [range]).

**Results:** Hypertension was present in 67 and 75% of patients at T_1_ and T_2_, respectively. Controlled hypertension was documented in 37 and 44% by OBP and 40 and 43% by ABPM. Nocturnal hypertension was present in 35 and 30% at T_1_ and T_2_; 24 and 32% of the patients had masked hypertension, while white coat hypertension was present in 16 and 21% at T_1_ and T_2_, respectively. Blood pressure by ABPM correlated significantly with GFR and PWV at T_2_, while PWV also correlated significantly with T_2_ cholesterol levels. Patients with uncontrolled hypertension by ABPM had a significant decrease in GFR, although not significant with OBP. Anemia and increased HOMAi were present in ~20% of patients at T_1_ and T_2_.

**Conclusion:** Pediatric RTx patients harbor risk factors that may affect their cardiovascular health. While we were unable to predict the evolution of renal function based on PWV and ABPM at T_1_, these risk factors correlated closely with GFR at follow-up suggesting that control of hypertension may have an impact on the evolution of GFR.

## Introduction

Pediatric kidney transplant recipients (RTx) have a decreased life expectancy. Although recent studies have shown an improvement in long term survival after RTx, it remains 20–25 years shorter compared to the general population (1). Cardiovascular disease (CVD) is the leading cause of mortality, accounting for 22–36% of all deaths (2, 3).

Hypertension (HT) is a common condition in RTx, with a prevalence reaching up to 80% (4). While major cardiovascular (CV) events are rare in RTx, changes in vascular wall structure may nonetheless already be present in early stages of chronic kidney disease (CKD). Previous studies have confirmed a correlation between the severity of hypertension and cardiac morbidity as well as the evolution of graft function, suggesting that adequate control of blood pressure could improve cardiovascular health and kidney graft survival both in adults and children (4–6).

Twenty-four hour ambulatory blood pressure monitoring (ABPM) is the most suitable method for the diagnosis and follow-up of hypertension, as it can identify masked [elevated blood pressure (BP) occurring outside the clinical setting] and isolated nocturnal hypertension (hypertension during sleep only), as well as blunting of the normal nocturnal dip (6). Both nocturnal hypertension and non-dipping is associated with an elevated risk of CV events in adults (7–9), as well as in children (10–12).

Pulse wave velocity (PWV) is a validated marker of vascular damage in adult CKD patients, and a predictor of CV events. Such link is yet to be established in children. Elevated arterial stiffness is a common finding in pediatric end-stage renal disease (ESRD) patients (13, 14), contributing to overall morbidity and mortality.

The aims of the present study were to (i) assess the prevalence of hypertension as well as the presence of known metabolic CV risk factors and of increased arterial stiffness in a pediatric RTx cohort, (ii) describe longitudinal changes in ABPM and PWV, and (iii) identify associations between blood pressure, arterial stiffness, and graft function.

## Patients and Methods

### Patients and Study Design

Of the eighty-seven patients controlled at our outpatient clinic, 52 RTx were available for the study. Inclusion criteria were a functioning allograft, with stable kidney function, without rejection episodes for at least 6 months prior to testing, absence of history of overt cardiovascular disease and an evaluable data set for both the first and second follow-up examinations.

Patients were assessed twice. A first cross-sectional checkup (T_1_) was followed by a second after ~6 years follow-up (T_2_). At both visits, standard yearly examinations were performed including evaluation of biometric data, laboratory tests, ABPM and PWV measurements (see below for details). Doppler renal ultrasound was part of the annual assessment and no renal artery stenosis was observed.

### Methods

Biometric data, etiology of ESRD, history of dialysis and transplantation, as well as immunosuppressive and antihypertensive medications were collected from medical charts. Height, weight, and BMI Z-scores were calculated using the CDC/WHO growth charts with overweight being defined as BMI >95 percentile (15, 16).

Laboratory data including creatinine, hemoglobin (Hgb), serum calcium (Ca), and phosphate (P), insulin resistance characterized by the HOMA index, as well as lipid profile at the time of follow-up were retrieved for analysis from the medical records.

HOMA index was calculated as fasting insulin multiplied by fasting glucose level divided by 22.5, as described by Matthews et al. (17). A cut-off level of >2.83 was used as described by Tresaco et al. (18). Diabetes mellitus (DM) was defined as a fasting glucose level >7 mmol/l or a 2-h plasma glucose level >11.1 mmol/l, based on the 2006 WHO criteria (19).

The revised Schwartz formula was used to calculate the glomerular filtration rate (GFR) (20). Proteinuria was defined as a urine protein/creatinine ratio greater than 200 mg/g (21).

### Blood Pressure Measurements

Brachial blood pressure and heart rate were measured by a validated automated oscillometric device (Omron M4, Omron Healthcare, Kyoto, Japan) in sitting position using appropriate cuff sizes, with the mean of three measurements being used for analysis. OBP results are presented as absolute values and height-based Z-scores (22).

ABPM was performed by using a validated automated oscillometric device (ABPM-04, Meditech Kft., Budapest, Hungary) (23). BP was measured at 20-min intervals during the day, and every 30 min during the night (23, 24). The mean ambulatory BP for wake, sleep and 24-h cycles and the nocturnal dip were calculated according the 2017 guidelines (25, 26). ABPM data were expressed as Z-score for sex and height (27). Hypertension was defined as SBP and/or DBP equal or exceeding the 95th percentile for gender, height, or the use of antihypertensive medication.

Hypertensive children were further classified according to the control of hypertension. Uncontrolled hypertension was defined as SBP and/or DBP values exceeding the 95th percentile for height in patients with or without antihypertensive medication. In patients with controlled hypertension, both SBP and DBP were below the 95th percentile for height and patients taking antihypertensive medication. The normotensive group included patients with SBP or DBP below the 95th percentile without taking antihypertensive medication.

Nocturnal dipping was defined as a nocturnal decrease in BP measured by ABPM. Patients with dipping below 10% were considered as non-dippers (25, 27).

Patients with BP exceeding 95th percentile at night, but with normal blood pressure during the day were categorized as having isolated nocturnal hypertension (28, 29).

Masked hypertensives had normotensive OBP values, but had hypertension on ABPM, whereas patients with white coat hypertension had elevated OBP but had normotension on ABPM (30).

### PWV Measurement

Carotid-femoral PWV measurement was performed by applanation tonometry with a PulsePen®™ (DiaTecne, Milan, Italy) device, as described previously (31). All participants were assessed in supine position. Arterial path length was determined by surface measurement, by subtracting the suprasternal-notch to carotid site distance from the suprasternal-notch to femoral site distance (31, 32). Aortic PWV was calculated as the distance of the carotid and femoral sampling sites divided by the time difference between the rise delay of the distal and proximal pulse according to the R wave belonging to the ECG qRs complex. Age-, sex-, and height-specific Z-scores were calculated using our previously established normative data (33).

### Statistical Analysis

Statistical analyses were performed using IBM SPSS 26. Age is expressed as median and interquartile ranges. Continuous variables, reported as means and standard deviations, were compared with the Wilcoxon signed-rank test. Categorical variables were compared using McNemar's test. The Mann-Whitney test was used for comparing groups in the cohort.

Correlations between variables were assessed by linear regression analysis. A *p*-value of <0.05 was considered statistically significant.

## Results

### Study Population

A total of 52 pediatric and young adult kidney transplant recipients (22 males) were included in the study. The median age [IQR] at T_1_ and T_2_ was 13.6 [11.1–16.2] years and 18.9 [16.8–23.9] years, respectively, with a follow-up of 5.7 [4.6–9.3] years.

Etiologies of kidney disease were congenital anomalies of the kidney and urinary tract (*n* = 13; 25%); focal segmental glomerulosclerosis (FSGS) (*n* = 11; 21%) (all patients had a genetically confirmed podocyte mutation); cystic kidney disease (*n* = 7, 13%); glomerulopathy (*n* = 5, 10%); nephronophtisis (*n* = 5; 10%); interstitial nephritis (3; 6%); acute tubular necrosis (*n* = 1; 2%); nephrocalcinosis (*n* = 1; 2%); Bardet-Biedl syndrome (*n* = 1; 2%); Denys-Drash syndrome (*n* = 1; 2%); cystinosis (*n* = 1; 2%); and unknown (*n* = 3; 6%).

Transplant recipients were on standard immunosuppression therapy with a calcineurin inhibitor (CNI) (tacrolimus or cyclosporine A) and mycophenolate mofetil. The dose of CNI inhibitors was adjusted to be in the target range and all patients were in the range at the time of the examinations. In addition, 60% of the patients were taking steroids at T_1_ and 44% at T_2_.

Patient characteristics at T_1_ and T_2_ are detailed in Table 1.

**Table 1:** Patient characteristics at T_1_ and T_2._.

	**T**_**1**_		**T**_**2**_		***p*-value**
Female	30	58%			
Male	22	42%			
Follow-up [years]	–		5.7	[4.6–9.3]	
Age [years]	13.6	[11.1–16.2]	18.89	[16.8–24.0]	* <0.001
Age at time of transplantation [years]	10.8	[8.5–12.7]			
Time since transplantation [years]	2.4	[1.0–4.7]	9.3	[6.3–11.8]	* <0.001
Cumulative time on dialysis [months]	11.0	[5.2–20.6]			
Number of second transplantations	5	10%			
Cadaver donor	46	88%			
Living related donor	6	12%			
Preemptive transplantation	9	17%			
CAPD	29	56%			
HD	9	17%			
CAPD & HD	5	10%			
Height Z score	−0.92	±1.39	−0.68	±1.59	0.06
Height <5pc	17	33%	12	23%	0.166
Weight Z score	−0.02	±1.17	−0.03	±1.41	0.9
BMI Z score	0.39	±0.88	0.48	±0.99	0.47

*Continuous variables are presented as mean ± SD, time parameters are shown as median [IQR], categorical variables are expressed as number [percentage]*.

*T_1_, first follow-up visit; T_2_, second follow-up visit; BMI, body mass index; CAPD, continuous ambulatory peritoneal dialysis; HD, hemodialysis; 5pc, 5th percentile*.

* *Significant p-values are indicated with an asterisk*.

A trend in catch-up growth could be observed during the course of the study (*p* < 0.06), with no significant difference in weight and BMI-Z scores between T_1_ and T_2_. Of note, while there was an almost −1 SD deficit in height Z score at T_1_, patient weight was appropriate for age, with a positive BMI Z score at both T_1_ and T_2_. Overweight was present in 4 (7.6%) and 6 (11.5%) patients at T_1_ and T_2_, respectively.

Relevant laboratory results are presented in Table 2.

**Table 2:** Laboratory data at T_1_ and T_2_.

	**T_**1**_**		**T_**2**_**		***p*-value**
GFR (ml/min/1.73 m^2^)	62.1	±30.7	60.4	±34.4	NS
GFR <60	25	48%	26	50%	NS
Hemoglobin (g/l)	129.2	±16.5	133.5	±27.3	NS
Hemoglobin <100	10	19%	10	19%	
Diabetes	3	6%	3	6%	NS
HOMA index >2.8	10	19%	10	19%	NS
Cholesterol (mmol/l)	4.32	±1.98	4.56	±1.71	0.06
Cholesterol >5.2	6	12%	11	21%	NS
Triglycerides (mmol/l)	1.56	±1.13	1.44	±1.32	NS
Triglycerides >1.1 mmol/l	24	46%	16	30%	NS
Proteinuria (#)	8	15%	19	36%	*0.007

*Continuous variables are presented as mean ± SD. Categorical variables are expressed as number (percentage)*.

*T_1_, first follow-up visit; T_2_, second follow-up visit; GFR, glomerular filtration rate; HOMA, Homeostasis Model Assessment - Insulin Resistance; NS, not significant*.

* *Significant p-values are indicated with an asterisk*.

# *Urine protein/creatinine ratio 50–200 mg/mmol*.

GFR did not change significantly during follow-up. Approximately half of the patients had a GFR below 60 ml/min/1.73 m^2^ at both time points. Anemia was present in 19 and 20% at T_1_ and T_2_, respectively. Nineteen percent of the patients had an increased HOMA index. Three patients had diabetes and a considerable proportion of patients had abnormal serum lipid levels. Proteinuria was present in 15 and 36% at T_1_ and T_2_, respectively; however, none of the patients had nephrotic range proteinuria at T_1_ or T_2_.

No abnormal values were observed in calcium and phosphate metabolism at the time of the study.

### Antihypertensive Medication

Antihypertensive medication consisted of calcium-channel blockers (CCB) (T_1_: *n* = 27; T_2_: *n* = 25), beta-blockers (BB) (T_1_: *n* = 21; T_2_: *n* = 24), ACE-inhibitors (ACEi) or angiotensin receptor blockers (ARB) (T_1_: *n* = 9; T_2_: *n* = 17), diuretics (thiazide or indapamide) and alpha-adrenergic blocking agents (T_1_: *n* = 6; T_2_: *n* = 10). Mean number of antihypertensive medication was 1.3 ± 1.2 at T_1_ and 1.5 ± 1.2 at T_2_ (*p* = NS).

### Prevalence of Hypertension According to Office and ABPM Categories

All 52 patients had their OBP measurements recorded, while ABPM results were available for 37 patients. The prevalence of previously diagnosed hypertension in the whole cohort was 35 (67%) and 39 (75%) at T_1_ and T_2_, respectively (*p* = NS). Controlled hypertension based on OBP measurements was 19 (37%) and 23 (44%) at T_1_ and T_2_ (*p* = NS) (Table 3).

**Table 3:** Blood pressure follow-up based on office blood pressures in the whole cohort (*n* = 52), and ABPM measurements (*n* = 37).

	**Office BP**				**ABPM**					**Discordance between Office and ABPM results**			
	**T**_**1**_		**T**_**2**_		**T**_**1**_		**T**_**2**_			**T**_**1**_		**T**_**2**_	
	***n***	**%**	***n***	**%**	***n***	**%**	***n***	**%**		***n***	**%**	***n***	**%**
Untreated HT	2	4%	3	6%	3	8%	0	0%	White coat HT	6	16%	8	21%
Uncontrolled HT	16	31%	16	31%	11	30%	13	35%	Masked HT	9	24%	12	32%
Controlled HT	19	36%	23	44%	15	40%	18	49%	**ABPM categories**				
Normotension	15	29%	10	19%	8	22%	6	16%	24 h HT	23	62%	25	67%
									Daytime HT	5	14%	7	19%
									Isolated nocturnal HT	9	24%	5	14%

*T_1_, first follow-up visit; T_2_, second follow-up visit; ABPM, 24 h ambulatory blood pressure monitoring; HT, hypertension*.

There was no significant difference between the OBP values of the whole cohort (*n* = 52) and those who also had ABPM measurements (*n* = 37) (data not shown).

Among those who had ABPM results, 26 (70%) and 31 (84%) had hypertension at T_1_ and T_2_, respectively (*p* = NS). Controlled hypertension based on ABPM was present in 15 (40%) and 18 (49%) patients at T_1_ and T_2_, respectively (*p* = NS).

Of those who had controlled hypertension at the first measurement, 9 (60%) were non-dippers. while this ratio was 7 (39%) at T_2_ (*p* = NS). The prevalence of non-dippers among uncontrolled hypertensives was 9 (81%) at T_1_, and 8 (61%) at T_2_ (*p* = NS), respectively. Isolated daytime hypertension was present in 5 (14%) and 7 (19%) cases, whereas isolated nocturnal hypertension was present in 9 (24%) and 5 (14%) of cases at T_1_ and T_2_, respectively.

Using both ABPM and office results, 9 (24%) and 12 (32%) patients had masked hypertension, while white coat hypertension was present in 6 (16%) and 8 (21%) patients at T_1_ and T_2_, respectively.

Details relative to blood pressure and PWV results are shown in Tables 4A,B, respectively.

**Table 4A:** Blood pressure data at T_1_ and T_2_.

	**T**_**1**_		**T**_**2**_		***p*-value**
Office SBP	122	±14	126	±15	0.12
Office SBP-Z	0.92	±1.7	1.09	±2.0	0.83
Office DBP	74	±9	77	±11	*0.03
Office DBP-Z	0.36	±1.55	0.82	±1.85	0.075
24 h SBP	119	±11	123	±14	0.19
24 h SBP-Z	1.35	±1.40	1.28	±1.93	0.38
24 h DBP	68	±7	71	±11	0.18
24 h DBP-Z	0.38	±1.18	0.7	±2.05	0.90
Daytime SBP	123	±11	125	±16	0.49
Daytime SBP-Z	1.12	±1.30	0.91	±2.16	0.64
Daytime DBP	71	±7	74	±10	0.25
Daytime DBP-Z	−0.04	±1.21	0.13	±1.77	0.92
Nighttime SBP	113	±13	116	±16	0.20
Nighttime SBP-Z	1.72	± 1.44	1.73	±2.15	0.41
Nighttime DBP	61	±13	65	±12	0.15
Nighttime DBP-Z	1.07	± 1.91	1.42	±2.03	0.59

*Office blood pressure: n = 52; ABPM: N = 37*.

*Continuous variables are presented as mean ± SD*.

*T_1_, first follow-up visit; T_2_, second follow-up visit; ABPM, 24 h ambulatory blood pressure monitoring; SBP, systolic blood pressure; DBP, diastolic blood pressure; SBP-Z, systolic blood pressure Z score; DBP-Z, diastolic blood pressure Z score*.

* *Significant p-values are indicated with an asterisk*.

**Table 4B:** Results of PWV measurements.

	**T**_**1**_		**T**_**2**_		***p*-value**
PWV absolute value	5.39	±0.9	5.82	±1.175	*0.03
PWV-Z	0.519	±1.041	0.40	±1.29	0.373

*Continuous variables are presented as mean ± SD. PWV-Z, pulse wave velocity Z score*.

* *Significant p-values are indicated with an asterisk*.

### Blood Pressure

There was no significant change between T_1_ and T_2_ in either OBP or ABPM blood pressure Z-scores.

### PWV Results

All children had carotid-femoral PWV measurements performed at both follow-up visits (Table 4B). While the absolute value of PWV increased significantly, there was no difference in Z scores at T_1_ and T_2_ (Table 4B).

### Correlations

Correlations between blood pressure, GFR, PWV as well as blood pressure control and evolution of GFR and presented in Tables 5A–C.

**Table 5A:** Correlation between T_2_ GFR and T_2_ ABPM.

	***R***	***p*-value**
**T**_**2**_ **GFR**		
T_2_ 24h SBP-Z	0.562	*0.0001
T_2_ 24h DBP-Z	0.444	*0.007

* *Significant p-values are indicated with an asterisk*.

**Table 5B:** Correlation between T_2_ PWV-Z and T_2_ ABPM.

	**R**	***p*-value**
**T**_**2**_ **PWV-Z**		
T_2_ 24h SBP-Z	0.437	*0.009
T_2_ 24h DBP-Z	0.523	*0.001

* *Significant p-values are indicated with an asterisk*.

**Table 5C:** Comparison of renal function outcomes between controlled and uncontrolled hypertensives.

	**Controlled**	**Uncontrolled**	***p*-value**
**ΔGFR** expressed in ml/min/1.73m^2^			
Office BP	1.55 ± 23.0	−2.4 ± 31.2	0.52
ABPM BP	3.0 ± 21.2	−14.3 ± 20.1	*0.03

*Continuous variables are presented as mean ± SD*.

T_1_, first follow-up visit; T_2_, second follow-up visit; GFR, glomerular filtration rate; SBP-Z, systolic blood pressure Z score; DBP-Z, diastolic blood pressure Z score; PWV-Z, Pulse wave velocity Z score; ΔGFR, change of GFR from T_1_ to T_2_; ABPM, 24 h ambulatory blood pressure monitoring.

* *Significant p-values are indicated with an asterisk*.

### Blood Pressure and GFR

**Figure 1:**
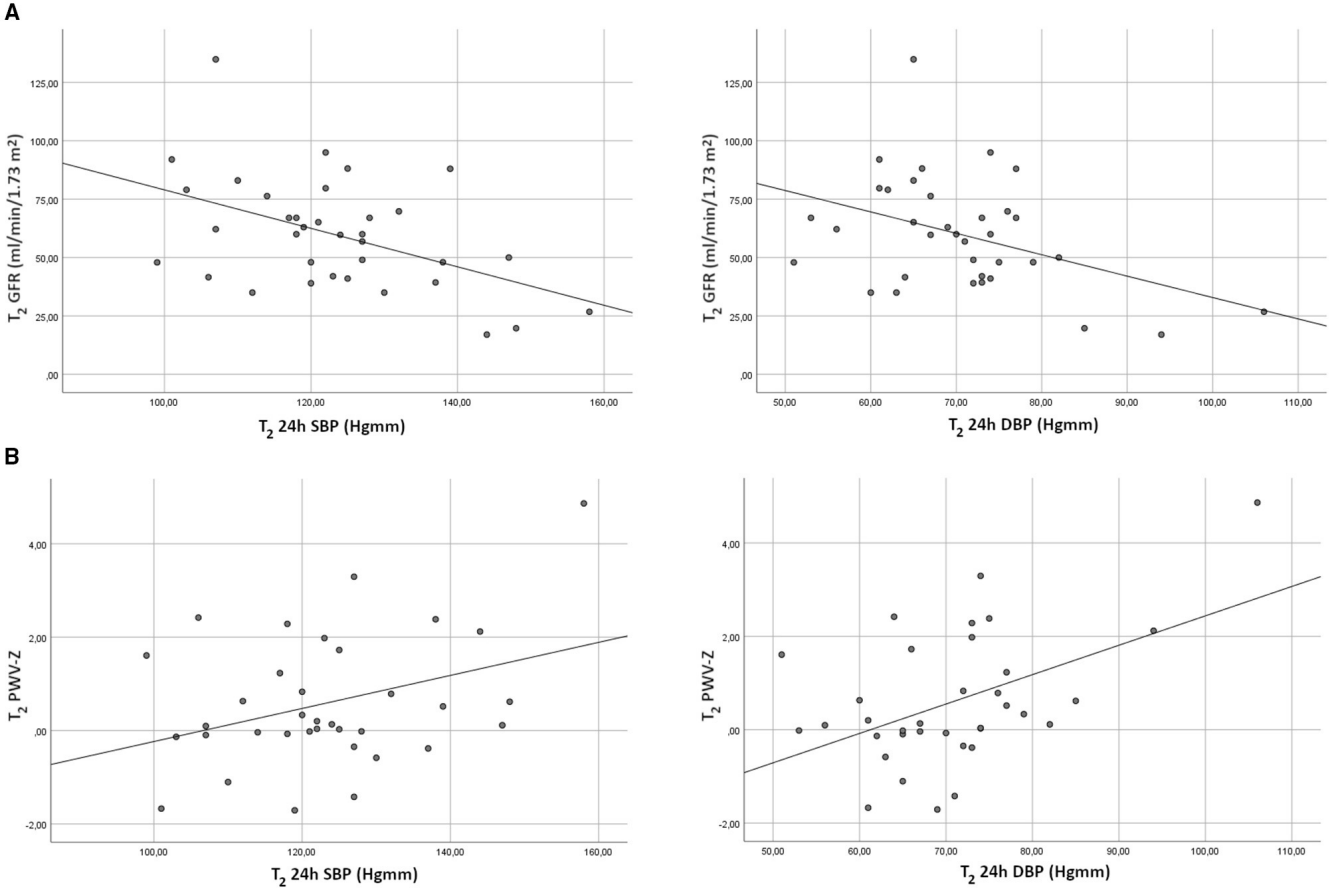
**(A)** Correlation of 24 h ABPM blood pressure values and GFR at the second follow-up. GFR, glomerular filtration rate; SBP, systolic blood pressure; DBP, diastolic blood pressure. **(B)** Correlation of 24 h ABPM blood pressure values and PWV-Z at the second follow-up. PWV, pulse wave velocity; SBP, systolic blood pressure; DBP, diastolic blood pressure.

There was no correlation between ABPM blood pressure measurements at T_1_ and GFR values either at T_1_ or at T_2_. In contrast, all systolic and diastolic ABPM Z scores were closely correlated with GFR at T_2_ (shown graphically in Figure 1A).

Dipper status did not affect kidney function. Of note, OBP values (either for the whole cohort or those with accompanying ABPM results) did not correlate with GFR whether at T_1_ or at T_2_.

### Arterial Stiffness by PWV

There was no correlation between any of the blood pressure values (office or ABPM) and PWV-Z at T_1_. However, there was a positive correlation between T_2_ PWV-Z and all systolic and diastolic ABPM-Z scores (data shown for 24 h systolic and diastolic values), while office blood-pressure Z scores showed no correlation with PWV-Z at T_2_ (shown graphically in Figure 1B).

There was no correlation between GFR and PWV-Z values. Regarding lipid measurements, T_2_ PWV correlated with T_2_ cholesterol (*R* = 0.619, *p* < 0.001).

### Blood Pressure Control and GFR

**Figure 2:**
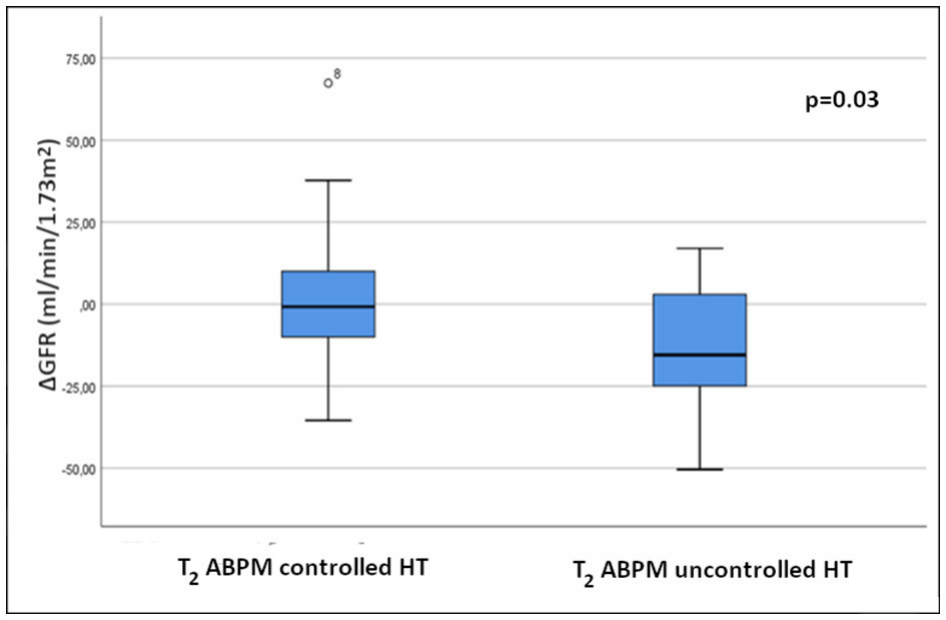
Comparison of changes in GFR between controlled and uncontrolled hypertensive patients at the second follow-up. ΔGFR, GFR difference between T2 and T1 (T2 _GFR_ - T1_GFR_).

While there was no significant difference between GFR values of patients with controlled and those with uncontrolled hypertension at the first follow-up, patients with uncontrolled hypertension at T_2_ had a significant decrease in GFR (shown as change in GFR between T_1_ and T_2_) compared to controlled hypertensives. This difference was only present if ABPM values were considered, and not for OBP (shown graphically in Figure 2).

## Discussion

Functional and structural arterial damage is already present in children with CKD, along with an increased risk of cardiovascular morbidity (6, 34–36). It has also been shown that RTx decreases the risk of CV, although remains approximately two magnitudes higher than in the normal population (5, 36, 37). In contrast to adults, hard endpoints of CV events are rare in RTx children, thus data are needed to establish the presence of cardiovascular risk factors and to assess the value of the various non-invasive measurements of cardiovascular health.

In this follow-up study, in addition to anthropometric data, we assessed the presence of several metabolic risk factors, the prevalence of hypertension and increased arterial stiffness and decreased GFR to identify associations and longitudinal changes in a pediatric RTx population.

Our patients exhibited some growth deficit with a trend of catch-up growth observed during follow-up. The reason for the substantial growth deficit reported in earlier studies (38) comparatively to our patients' growth delay (averaging around −1 SD) may be that all patients with CKD in the current study were on growth hormone treatment prior to transplantation. Since GH was discontinued following RTx, the catch-up in height was the result of Tx rather than due to pharmacological therapy with growth hormone. Furthermore, both weight and BMI Z scores were in the normal range at both follow-up visits, which may be the result of proper dialysis treatment, regular dietary counseling, and control (39).

GFR remained at ~60 ml/min/173 m^2^ and did not deteriorate significantly during follow-up. However, approximately half of the patients had a GFR below 60 ml/min/1.73 m^2^ on both visits.

The prevalence of risk factors such as anemia, diabetes and insulin resistance did not change significantly during follow-up. There was a trend toward an increase in total cholesterol and a significant increase in non-nephrotic range proteinuria in our cohort. Such correlation between cholesterol and PWV at T_2_ is moreover in line with our previous report confirming that, after a median 2-year follow-up of renal transplant recipients, the correlation between cholesterol and PWV becomes significant (40).

### Prevalence of Hypertension: Controlled—Uncontrolled

The relatively high rate of uncontrolled hypertension with both office and ABPM measurements in the present study population despite close clinical follow-up and personalized antihypertensive treatment was rather unexpected although in keeping with previous reports (41–43). In addition, with ABPM, we were able to confirm a high proportion of masked and white coat hypertension, as well as nocturnal and isolated nocturnal hypertension. Furthermore, the absence of nocturnal blood pressure dipping was high among controlled and even higher among uncontrolled hypertensive. These findings are similar to previous studies (9, 40, 44) showing the superiority of ABPM over conventional BP measurement techniques (4, 28–30).

The causes behind the relatively high rates of uncontrolled hypertension are multifactorial (3–5, 7, 28–30, 37). Kidney transplants may show decreased GFR, and in fact, more than 50% of patients in our cohort had GFR values below 60. Immunosuppressants, namely CNIs and corticosteroids, may also contribute to increased blood pressure. However, our patients were within the target range for CNI values in both exams, and the steroid dose was minimized (2–4 mg methyprednisolone/day), with 40 and 56% of patients no longer receiving steroids at T1 and T2, respectively (see results, study population). We could not find any correlations between blood pressure, CNI levels or steroid consumption in the data analysis.

Adherence to blood pressure lowering medications is also an important issue. The study protocol did not include direct assessment of compliance, the fact that immunosuppressive drugs were in target range may indicate good adherence to antihypertensive therapy as well.

Finally, another factor contributing to hypertension could be recurrence of primary renal disease or post transplant glomerulopathy. Routine biopsies for screening these pathologies are not part of the protocol in our center. Since no clinical signs of *de novo* or recurrent glomerular disease was observed, we believe this may not be relevant to our study population.

### Evolution of Arterial Stiffness

While the absolute value of PWV increased significantly, the height-controlled Z-score remained unchanged, highlighting the necessity to use appropriate, height-controlled Z-scores for comparison purposes in children (45, 46).

One of the major purposes of assessing *surrogate markers* of vascular health is not only to confirm changes but also to predict subsequent cardiovascular hard endpoints. ABPM-measured blood pressure and PWV are established markers and cardiovascular risk factors according to adult studies (47, 48). In the current assessment, neither blood pressure nor PWV at T_1_ were able to anticipate the evolution of GFR during follow-up. However, there was a close correlation between arterial stiffness as well as ABPM blood pressure and GFR at T_2_. In addition, uncontrolled hypertensives (by ABPM) exhibited a significant decrease in GFR at T_2_ compared to the controlled group thus suggesting that controlling hypertension may impact the evolution of GFR on the long term (49–51). Once again, blood pressure measurement by ABPM revealed to be superior to OBP values since OBP values (whether in the whole cohort or in those with both office and ABPM results) did not correlate with GFR at T_2_.

### Limitations

This single-center follow-up study has some important limitations due to the relatively low sample size, related to the low prevalence of ESRD and transplantation in children. This limited availability also determines the limits of statistical analysis. Although followed at a regional transplant center, not all patients could be included in the study, hence the results are not unreservedly applicable to the entire RTx population. Furthermore, only a portion of the whole cohort had ABPM results. However, given that the OBP values of the ABPM sub-study did not differ from the entire cohort and that these OBP results failed to show the correlations observed with ABPM, we can still affirm the superiority of ABPM over OBP. Since the proteinuria was assessed semiquantitatively, no correlations could be calculated.

The fact that dipper status did not affect kidney function may be explained by the small number of patients in each category, which may be too low to reveal differences. This is also true for the subgroups of isolated nocturnal hypertension and white coat hypertension. Finally, the correlations found between ABPM, PWV, and GFR are not necessarily causal since decreasing GFR may also be the cause of uncontrolled hypertension and vice-versa.

## Conclusion

In conclusion, our study provides additional data on the general CV health of RTx children more than 2 and 9 years after transplantation. Pediatric RTx patients harbor several cardiovascular risk factors that may affect their cardiovascular health. While we were not able to predict the evolution of renal function using surrogate markers such as PWV and ABPM blood pressure at T_1_, these risk factors were closely correlated with renal function at follow-up, with control of hypertension having a significant impact on GFR evolution.

## Data Availability Statement

The raw data supporting the conclusions of this article will be made available by the authors, without undue reservation.

## Ethics Statement

The studies involving human participants were reviewed and approved by Semmelweis University Regional and Institutional Committee of Science and Research Ethics (TUKEB 91/4-2008). Written informed consent to participate in this study was provided by the participants' legal guardian/next of kin.

## Author Contributions

AV and AB organized the database performed the statistical analysis and wrote the first draft of the manuscript. OC, ÉK, KK, and ST wrote sections of the manuscript. AS and GR contributed to conception and design of the study and corrected the manuscript. All authors read and approved the submitted version.

## Funding

This study was supported by the Hungarian National Research, Development and Innovation Office grants NKFI-124549 (GR) and TK2121GYKI (AS), and the Research Group of the Hungarian Academy of Sciences at the First Department of Pediatrics (AS).

## Conflict of Interest

The authors declare that the research was conducted in the absence of any commercial or financial relationships that could be construed as a potential conflict of interest.

## Publisher's Note

All claims expressed in this article are solely those of the authors and do not necessarily represent those of their affiliated organizations, or those of the publisher, the editors and the reviewers. Any product that may be evaluated in this article, or claim that may be made by its manufacturer, is not guaranteed or endorsed by the publisher.
